# 85. Evaluation of Urinalysis and Urine Culture Use at a Community Health-system

**DOI:** 10.1093/ofid/ofab466.287

**Published:** 2021-12-04

**Authors:** Martin Brenneman, Brian C Bohn, Sarah E Moore, Ashley Wilde, Ashley Wilde, Matthew Song

**Affiliations:** Norton Healthcare, Louisville, Kentucky

## Abstract

**Background:**

The Infectious Diseases Society of America asymptomatic bacteriuria (ASB) guidelines recommend against screening for or treating ASB in most patients without symptoms of a urinary tract infection (UTI). The purpose of this study was to characterize current urine testing practices and their potential impact on identification and treatment of asymptomatic bacteriuria on hospitalized adults.

**Methods:**

This retrospective, point prevalence study conducted at a 4 hospital community health-system that included all inpatients ≥ 18 years old present on November 13^th^, 2019. Patients were excluded if they were admitted or transferred to either a labor & delivery or mother-baby unit. A chart review was performed for a sub-group of patients with abnormal urine testing, with a target sample size of 200 (n=50 from each hospital). The primary outcome was the prevalence of patients with a urinalysis, urine culture, or both performed during their admission. Secondary outcomes included abnormal urine testing in the overall cohort and symptomatology and antibiotic use in the sub-group (Figure 1).

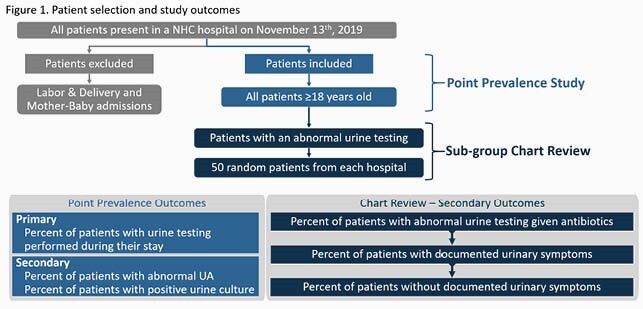

**Results:**

947 patients met inclusion criteria. Of those patients, 516 (54%) had urine testing performed during their admission. 322 (34%) patients had abnormal urine testing results (Table 1). In the sub-group, 192 patients with abnormal urine tests were included. Antibiotics with a documented indication of UTI were administered to 66 (34%) patients. Of those given antibiotics with a UTI indication, 49/66 (74%) did not have documented signs or symptoms of a UTI (Figure 2).

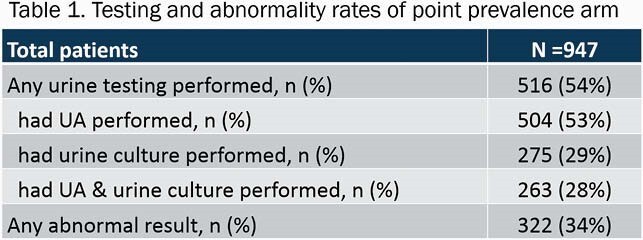

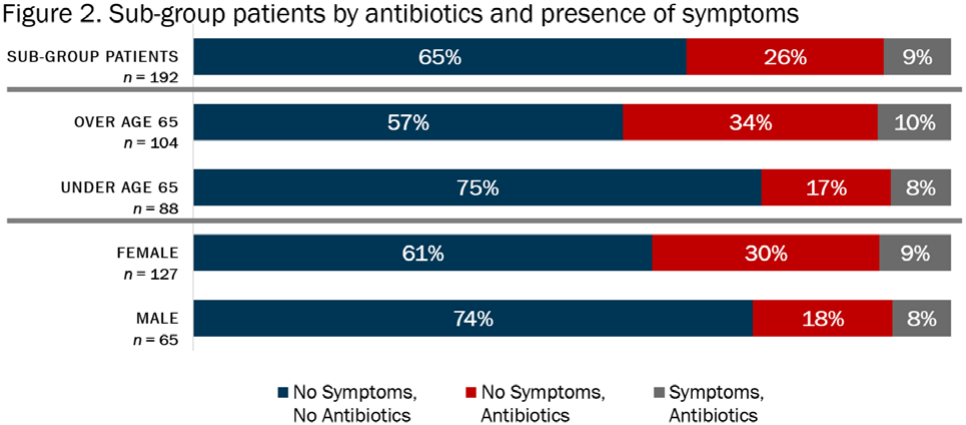

**Conclusion:**

Urine testing was performed on the majority of admitted adult patients. Unnecessary testing likely contributes to guideline discordant screening and treatment of ASB. Future studies are needed to identify effective diagnostic stewardship interventions to decrease screening and treatment of ASB.

**Disclosures:**

**Ashley Wilde, PharmD, BCPS-AQ ID**, Nothing to disclose

